# Malaria burden in a birth cohort of HIV-exposed uninfected Ugandan infants living in a high malaria transmission setting

**DOI:** 10.1186/s12936-016-1568-z

**Published:** 2016-10-18

**Authors:** Abel Kakuru, Paul Natureeba, Mary K. Muhindo, Tamara D. Clark, Diane V. Havlir, Deborah Cohan, Grant Dorsey, Moses R. Kamya, Theodore Ruel

**Affiliations:** 1 Infectious Diseases Research Collaboration, Kampala, Uganda; 2 Department of Medicine, University of California, San Francisco, USA; 3 Department of Obstetrics and Gynecology, University of California, San Francisco, USA; 4 Department of Medicine, Makerere University College of Health Sciences, Kampala, Uganda; 5 Department of Pediatrics, University of California, San Francisco, USA

**Keywords:** Malaria, HIV-exposed uninfected infants, Placental malaria

## Abstract

**Background:**

HIV-exposed, uninfected (HEU) infants suffer high morbidity and mortality in the first year of life compared to HIV-unexposed, uninfected (HUU) infants, but accurate data on the contribution of malaria are limited.

**Methods:**

The incidence of febrile illnesses and malaria were evaluated in a birth cohort of HEU infants. Infants were prescribed daily trimethoprim–sulfamethoxazole (TS) prophylaxis from 6 weeks of age until exclusion of HIV-infection after cessation of breastfeeding. Infants were followed for all illnesses using passive surveillance and routine blood smears were done monthly. Malaria was diagnosed as a positive blood smear plus fever. Placental malaria was determined by histopathology, placental blood smear and PCR. Risk factors for time to first episode of malaria were assessed using a Cox proportional hazards model. Malaria incidence among HEU infants aged 6–12 months was compared to that in other cohorts of HEU and HUU infants from the same region.

**Results:**

Among 361 HEU infants enrolled, 248 completed 12 months of follow-up resulting in 1562 episodes of febrile illness and 253 episodes of malaria after 305 person-years of follow-up. The incidence of febrile illness was 5.12 episodes per person-year (PPY), ranging from 4.13 episodes PPY in the first 4 months of life to 5.71 episodes PPY between 5 and 12 months of age. The overall malaria incidence was 0.83 episodes per person-year (PPY), increasing from 0.03 episodes PPY in the first 2 months of life to 2.00 episodes PPY between 11 and 12 months of age. There were no episodes of complicated malaria. The prevalence of asymptomatic parasitaemia was 1.2 % (19 of 1568 routine smears positive). Infants born to mothers with parasites detected from placental blood smears were at higher risk of malaria (hazard ratio = 4.51, P < 0.001). HEU infants in this study had a 2.4- to 3.5-fold lower incidence of malaria compared to HUU infants in other cohort studies from the same area.

**Conclusion:**

The burden of malaria in this birth cohort of HEU infants living in a high-transmission setting and taking daily TS prophylaxis was relatively low. Alternative etiologies of fever should be considered in HEU-infants taking daily TS prophylaxis who present with fever.

*Trial Registration* NCT00993031, registered 8 October, 2009

## Background

As the number of infants born HIV-exposed but uninfected (HEU) steadily increases throughout Africa [1], it is important to understand their burden of malaria. Several early studies suggested that HEU African children suffer greater infectious disease morbidity and mortality than HIV-unexposed uninfected infants (HUU) [2–7], but these studies were performed when women had limited access to antiretroviral therapy (ART). The World Health Organization (WHO) currently recommends that all HIV-infected individuals, including pregnant women, should take ART, and HEU infants should receive daily trimethoprim-sulfamethoxazole (TS) prophylaxis from 6 weeks of age until the risk of HIV transmission ends and HIV infection is excluded [8]. TS has been shown to be effective in reducing the incidence of malaria among older children [9–12]. However, accurate data on the incidence of malaria among infants of HIV-infected mothers receiving ART and who, themselves receive TS, are needed to guide clinical management.

The objective of this study was to describe the natural history of malaria in a birth cohort of Ugandan HEU infants who were born to mothers who received ART as part of a clinical trial during pregnancy and who themselves received TS prophylaxis starting at ~6 weeks of life, as per current WHO guidelines. The incidence of malaria and all febrile illnesses and the prevalence of asymptomatic parasitaemia at routine visits were measured over the first year of life. Risk factors for malaria in the first year of life were assessed at the time of birth.

## Methods

### Study design, setting and population

This was a planned secondary data analysis in a birth cohort of HEU infants born to HIV-infected mothers who were part of a randomized controlled trial of lopinavir/ritonavir versus efavirenz-based ART living in Tororo district, Uganda [Protease Inhibitors to Reduce Malaria Morbidity in HIV-Infected Pregnant Women (PROMOTE-PIs), NCT00993031)] [13]. The study site is a high malaria transmission setting where transmission occurs year round with an average entomological inoculation rate of 310 infectious bites per person per year [14]. For this analysis, all infants who survived beyond the first 24 h of life and completed at least one visit to the study clinic after discharge from the hospital following birth were included. Gestational age was established using last menstrual period with confirmation by ultrasound [13]. Data from two other cohort studies conducted in the same region by the same researchers: Interactions Between HIV and Malaria in African Children (TCC, NCT00527800) [12] and Chemopreventive Therapy for Malaria in Ugandan Children (PROMOTE-Chemop, NCT00948896) [9], were included to enable comparisons with HUU infants and other HEU infants.

### Study participant follow-up

Infants were followed from birth to 1 year of age. At birth, placental malaria status was determined from placental blood and tissue. Infants were seen at monthly routine visits and parents were instructed to bring their infants to a dedicated study clinic, open 7 days a week, for any fever or other illness. All study participants were given a long-lasting, insecticide-treated bed net (LLIN) at birth and prescribed daily TS prophylaxis from 6 weeks of age until they were confirmed to be HIV negative after cessation of breastfeeding. At each monthly visit, adherence to TS prophylaxis was assessed by 3-day recall and parents were asked if infants were sleeping under an LLIN. A thick blood smear for determination of malaria parasitaemia by microscopy was performed at every routine monthly visit.

### Malaria diagnosis and treatment

A febrile episode was defined as having a measured tympanic temperature of ≥38.0 °C or a history fever in the past 24 h. Study participants who presented to the study clinic with a febrile episode had a thick blood smear done for the detection of malaria parasites. If the blood smear was positive, they were diagnosed with malaria and a thin blood smear was taken off for identification of parasite species. Infants with uncomplicated malaria were treated with artemether–lumefantrine (AL) (tablets of 20 mg of artemether and 120 mg of lumefantrine: Coartem, Novartis) if they were ≥4 months old and weighing ≥5 kg. Infants with uncomplicated malaria who were <4 months old and weighing <5 kg were treated with quinine.

### Laboratory methods

Thick and thin blood smears were stained with 2 % Giemsa and examined for malaria parasites by trained microscopists. A blood smear was considered negative when the examination of 100 high power fields did not reveal asexual parasites. All slides were read by a second reader, and a third reader settled any discrepancies. Placental specimens were collected within 30 min of delivery in the hospital (or as early as possible, if delivery occurred at home). Thick blood smears made from placental blood collected from an incision on the maternal surface of placental tissue, were examined for parasites. Aliquots of approximately 25 µL of placental blood were also placed on filter paper, air dried and stored for DNA extraction and PCR testing for malaria parasites as earlier described [15]. Placental tissues were processed for histological evidence of placental malaria as described previously [13]. Histological slides were read in duplicate by two trained independent readers, and the results were recorded on a standardized case-record form; any discrepant results were resolved by a third reader. The rate of inter-reader agreement was 71.3 % (kappa, 0.48; P < 0.001). The readers were unaware of both the treatment assignment and the results of previous reads.

### Statistical analysis

Data were double-entered into Access database (Microsoft, Redmond, WA, USA) and analysed using Stata version 12 (Stata Corp, College Station, TX, USA). Follow-up time started at birth and ended at 1 year of age, or the time of premature withdrawal, or when the study was stopped prior to reaching 12 months of age because of limited funding. An incident episode of malaria was defined as having a febrile episode with a positive blood smear not preceded by any treatment for malaria in the prior 14 days. Comparisons of the incidence of malaria between 6 and 12 months of age were made between infants enrolled in this study and other cohorts enrolled in the same study site using the same methodology. Placental malaria status was categorized using a categorical variable as follows: no parasites or pigment detected by any method, only pigment detected by histopathology (no parasites), parasites detected by PCR or histopathology but not placental blood smear, parasites detected by placental blood smear. Time to an infant’s first episode of malaria was estimated using Kaplan–Meier survival analysis. Associations between risk factors assessed at the time of birth and time to first episode of malaria were assessed using a Cox proportional hazards model. In all analyses, a two-sided P value of <0.05 was considered to be statistically significant.

### Ethical approval

This study was approved by the Uganda National Council of Science and Technology, the Makerere University School of Medicine Research Ethics Committee, and the University of California, San Francisco Committee for Human Research. Informed consent was obtained from all mothers at the time of enrolment.

## Results

### Study profile and subject characteristics

**Fig. 1:**
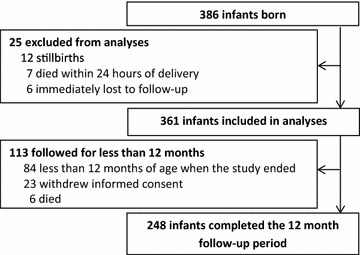
Study profile

Among 386 HEU infants born to 376 HIV-infected women (including ten pairs of twins), 361 infants were included in this analysis (Fig. 1). The median gestational age at birth was 38.9 weeks (range 29.7–43.0), including 63 (17.5 %) infants born pre-term (Table 1). Of the 361 infants included in the analysis, 248 reached 12 months of age. The majority of those followed for fewer than 12 months were due to premature study termination due to limited funds (Fig. 1). The median (range) follow-up time was 361 (16–365) days. Over the total of 305 person-years of follow-up time, infants were breastfed for 92 % and received TS for 91 % of the time. Among 361 children included, 350 were started on TS and 11 were withdrawn before TS could be started. Of these 350 children, only 31 stopped TS before they reached 1 year of age. Of the 31 who stopped TS before 1 year of age, 30 stopped only 2–63 days before reaching 1 year of age, and the remaining child stopped TS 114 days before reaching 1 year of age. At routine visits, TS adherence by self report was 98 %. Adherence to TS did not change as infants aged.

**Table 1:** Characteristics of study participants

Characteristic	Value (n = 361)^a^
*At birth*	
Female	167 (46.3 %)
Gestational age, weeks	38.9 (29.7–43.0)
Pre-term births^b^	63 (17.5 %)
Birth weight, kg	2.9 (1.3–4.4)
Low birth weight^c^	68 (19.4 %)
*Maternal HIV treatment arm*	
Efavirenz	178 (49.3 %)
Lopinavir/ritonavir	183 (50.7 %)
Mother diagnosed with malaria during pregnancy^d^	29 (8.0 %)
*Placental blood*	
Parasites by smear^e^	12 (4.0 %)
Parasites by PCR^f^	25 (18.6 %)
*Placental tissue histopathology* ^*g*^	
Parasites and pigment	14 (4.5 %)
Parasites only	7 (2.2 %)
Pigment only	81 (25.9 %)
No parasite or pigment	206 (66.0 %)
*During follow*-*up*	
Total follow-up time, person-years	305
Incidence of febrile illnesses, episodes per person-year	5.12
Incidence of malaria, episodes per person-year	0.83
Routine smear showed parasites (n = 1568 blood smears)^h^	19 (1.2 %)

^a^Data are n (%) or median (range) and apply to full cohort unless otherwise specified

^b^Pre-term birth: born at <37 weeks gestation

^c^Low birth weight: <2.5 kg

^d^One or more malaria episodes

^e^Among the 302 who had placental blood smears performed

^f^Among the 291 who had placental blood polymerase chain reaction (PCR) testing for malaria parasites performed

^g^Among the 312 who had placenta histopathology studies

^h^Among the 1568 routine smears examined

### Incidence of malaria and the prevalence of asymptomatic parasitaemia

**Fig. 2:**
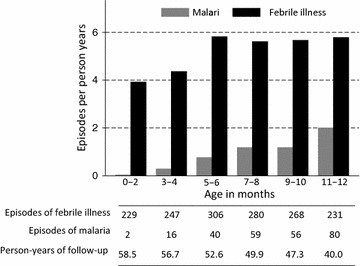
Incidence of febrile illnesses and malaria by age category

There were 1562 febrile episodes resulting in an overall incidence of 5.12 episodes per person year (PPY). Malaria was diagnosed in 253 (16.2 %) of the febrile episodes, resulting in an overall incidence of malaria of 0.83 malaria episodes PPY. The incidence of malaria steadily increased with age over the first year of life, rising from 0.03 episodes PPY in infants up to 2 months old to 2.00 episodes PPY in infants between 11 and 12 months old (Fig. 2). By contrast the incidence of febrile illnesses overall increased over the first 6 months of life and remained fairly stable from 6 to 12 months of age. Malaria episodes were treated with AL (n = 243) and quinine (n = 10). There were no episodes of complicated malaria or malaria-associated deaths. The incidence of malaria in HEU infants 6–12 months old and taking TS prophylaxis from this study was comparable to other studies of HEU receiving TS, and 2.4- to 3.5-fold lower than for HUU not receiving TS in the TCC and PROMOTE-Chemop studies (Table 2).

**Table 2:** Malaria burden in children 6–12 months of age living in Tororo, Uganda

Study	Mother’s HIV status	Prescribed TS prophylaxis	Date of observation	Number of children observed	Episodes of malaria	Person years of observation	Incidence of malaria PPY
TCC (NCT00527800)	Negative	No	Aug 2007–Oct 2008	99	157	42.8	3.67
	Positive	Yes	Aug 2007–Nov 2008	200	143	74.8	1.91
PROMOTE-Chemop (NCT00948896)	Negative	No	Jul 2010–Sep 2011	98	253	47.9	5.28
	Positive	Yes	Jul 2010–Mar 2012	197	111	72.4	1.53
PROMOTE PIs* (NCT00993031)	Positive	Yes	Sep 2010–Mar 2013	302	205	135.3	1.51

* Data from the present study

Asymptomatic parasitaemia, as determined from thick blood smears, was uncommon at routine visits. Among 1568 thick blood smears performed at monthly routine follow-up visits, 19 (1.2 %) were positive for malaria parasites among 16 different children; six of them developed malaria in the next 30 days, four had malaria during the previous 30 days, three had no further blood smears, and six became smear negative in the absence of anti-malarial therapy.

### Risk factors for time to first episode of malaria

**Fig. 3:**
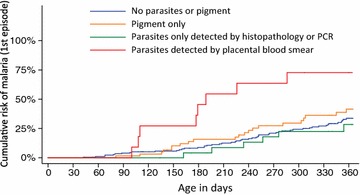
Risk of malaria over the first year of life, by placental malaria category

Overall, 108 children (29.9 %) developed at least one episode of malaria with a median time to their first episode of 225 days (range 42–357 days). Associations between risk factors assessed at birth and time to a first episode of malaria are presented in Table 3. Only placental malaria status was significantly associated with a higher risk of a first episode of malaria. Interestingly, compared to infants born to mothers with no evidence of placental malaria, infants born to mothers with parasites detected by placental blood smear had a higher risk of malaria in the first year of life (adjusted HR = 4.51, 95 % CI 2.10–9.68, P < 0.001), but not infants born to mothers with placental malaria based on the presence of pigment or parasites detected only by PCR or histopathology (Table 3; Fig. 3). Infants born with low birth weight had a lower risk of developing malaria in the first year of life but this did not reach statistical significance (adjusted HR = 0.55, 95 % CI 0.28–1.08, P = 0.08).

**Table 3:** Risk factors for time to a first episode of malaria

Variable	Categories	Number of infants	Univariate		Mutivariate	
			HR (95 % CI)	P value	HR (95 % CI)	P value
Gender	Male	160 (55.4 %)	Reference group		Reference group	
	Female	129 (44.6 %)	1.26 (0.83–1.93)	0.28	1.40 (0.91–2.17)	0.13
Mother’s ART regimen during pregnancy	Efavirenz-based	144 (49.8 %)	Reference group		Reference group	
	Lopinavir-based	145 (50.2 %)	1.23 (0.81–1.88)	0.33	1.21 (0.79–1.87)	0.38
Pre-term delivery	≥37 weeks	237 (82.0 %)	Reference group		Reference group	
	<37 weeks	52 (18.0 %)	1.02 (0.57–1.80)	0.96	1.28 (0.68–2.40)	0.45
Low birth weight	≥2500 g	233 (80.6 %)	Reference group		Reference group	
	<2500 g	56 (19.4 %)	0.70 (0.38–1.28)	0.25	0.55 (0.28–1.08)	0.08
Placental malaria status	No parasites or pigment	184 (63.7 %)	Reference group		Reference group	
	Pigment (not parasites) on histopathology	67 (23.2 %)	1.31 (0.79–2.18)	0.30	1.28 (0.76–2.17)	0.36
	Parasites only detected by PCR or histopathology	26 (9.0 %)	0.76 (0.33–1.78)	0.53	0.67 (0.29–1.58)	0.36
	Parasites detected by placental blood smear	12 (4.2 %)	3.98 (1.88–8.42)	<0.001	4.51 (2.10–9.68)	<0.001

Model includes the 289 participants who had data for these predictors

## Discussion

In this birth cohort of HEU infants living in an area of high transmission intensity, the burden of malaria was relatively low in the first year of life compared to HUU infants from the same area. Asymptomatic parasitaemia was rare and all episodes of malaria were uncomplicated, with a gradual increase in incidence from 2 to 12 months of age. Key characteristics of this birth cohort of HEU infants were the provision of LLINs, TS prophylaxis, ART for all mothers during pregnancy, and breastfeeding. These data fill an important gap in understanding the epidemiology of malaria in this vulnerable and growing population of HEU in sub-Saharan Africa, who are receiving the current standard of care, with TS prophylaxis from 6 weeks of life and being born to HIV-infected mothers who received ART. The most accurate data prior to this report came from the breastfeeding, antiretrovirals and Nutrition (BAN) trial, which reported a 48-week incidence of malaria of 0.052 episodes PPY [16]. However, the BAN trial took place in a clinical context that does not represent the current standard of care, because the women had not received ART during pregnancy and some of the infants (32 %) were born before TS prophylaxis had been introduced [16, 17].

As expected, malaria was very rare in HEU infants up to two months of age in this cohort. Relative protection from malaria in the first weeks of life has been well described among infants living in endemic areas [18], and is assumed to be a result of many factors, including transient protection from maternal anti-malarial antibodies that had been transferred in utero [19, 20], fetal haemoglobin [21], and riboflavin deficiency [22]. Beyond 2 months of age, the HEU infants in this cohort likely benefitted from the anti-malarial activity of TS, which has been well established by other studies [9, 10, 12, 17, 23]. The incidence of malaria of HEU infants aged 6–12 months in this cohort was over two to three times lower than that of HUU infants in prior studies (TCC study and PROMOTE Chemop trial) from the same endemic region [9, 10, 12]. While these comparisons are vulnerable to bias from the differences in the study time periods and populations, the lower incidence of malaria among HEU in this cohort was likely a result of the protective efficacy conferred by daily TS prophylaxis. The low prevalence of asymptomatic parasitaemia found in this study is also consistent with suppression by daily TS prophylaxis.

Active placental malaria by blood smear was associated with an increased risk of malaria over the first year of life in this cohort of HEU infants, as has been shown in other studies of HUU infants [24–27]. This association could simply be confounded by exposure; women with placental malaria likely have higher exposure to malaria and therefore infants born to them and living in the same households will also have a higher exposure to malaria [28]. But if that were the sole explanation, one would predict that infants whose placenta had evidence of infection from earlier in pregnancy (suggested by the presence of pigment) would also be at increased risk, but this was not so in this cohort. It is possible that exposure to malaria antigen earlier in pregnancy could confer protection to the fetus, counterbalancing the increased risk of exposure. Studies comparing the immune profiles of infants born with different in utero and placental exposure to malaria may shed light on this theory.

The contrasting pattern of the incidence of malaria versus febrile illness over the first year of life has clinical implications for the management of HEU infants living in malaria-endemic regions. In this cohort, the incidence of malaria rose from near zero in the first 2 months of life to two episodes PPY among infants between 11 and 12 months of age, while the incidence of febrile illness increased only slightly during the first 6 months of life and remained steady thereafter. Taken together, these patterns underscore the important use of accurate malaria diagnostics to evaluate all fevers in similar settings. Clinicians evaluating and treating febrile HEU infants, particularly those <2 months old, must not presume fever to represent malaria and should carefully evaluate for non-malarial causes of fever, such as bacterial sepsis and viral infections. Conversely, clinicians must suspect malaria in older febrile HEU infants, even if they are receiving TS. The highest incidence of malaria was seen in infants aged 11–12 months despite receiving daily TS prophylaxis. This could reflect waning protection from maternal antibodies or changes in TS adherence. In either case, the higher rates underscore the need for additional interventions to reduce malaria incidence in similar regions. Infants in this cohort had good clinical outcomes, with no episodes of complicated malaria or malaria-associated deaths among 253 episodes of malaria treated, but that is most likely a result of prompt diagnosis and treatment of malaria in the context of this trial and not available in the most limited resource clinical settings.

These results add to existing evidence that supports the importance of daily TS prophylaxis in preventing malaria in HEU children [9, 11, 12]. Recently presented data from Botswana have raised the question whether TS prophylaxis should be prescribed to all HEU living in Africa. The Mpepu study (NCT01229761) randomized HEU infants to receive TS or placebo from age 2–4 weeks until 15 months; the study was stopped because no mortality benefit was seen in the TS arm; the authors concluded that long term use of TS by HEU infants may not be indicated in settings where infants have low risk of HIV-transmission and malaria [29]. By contrast, our data suggest that in malaria endemic areas, TS can continue to play an important role in preventing morbidity and mortality from malaria among HEU infants.

This study had several limitations. The cohort’s sample size limited the power to evaluate additional risk factors for malaria. Comparisons of malaria incidence among infants who were 6–12 months old from other cohort studies were vulnerable to confounding by temporal differences in malaria transmission which were not adjusted for because long term longitudinal data about temporal changes in malaria transmission in the study area were not available. However, it is notable that the other cohorts were from the same study site and followed with identical malaria diagnostics and study protocols. Finally, there was very little follow-up time when infants were not taking TS and this limited our ability to separately investigate the effect of TS alone on malaria incidence.

## Conclusion

In this birth cohort of HEU infants living in a malaria-endemic region and taking daily TS prophylaxis, the incidence of malaria was relatively low but increased with increasing age. However, the burden of febrile illnesses was high and remained fairly stable during infancy. Alternative etiologies of febrile illnesses other than malaria should always be considered in HEU infants taking daily TS prophylaxis. While HEU infants living in malaria-endemic regions benefit from the malaria prophylactic efficacy of TS, additional strategies to reduce malaria in the important population are needed.
